# Efficacy and safety of immunosuppressive treatment in IgA nephropathy: a meta-analysis of randomized controlled trials

**DOI:** 10.1186/s12882-019-1519-3

**Published:** 2019-08-27

**Authors:** Zheng Zhang, Yue Yang, Shi-min Jiang, Wen-ge Li

**Affiliations:** 1 Department of Nephrology, China-Japan Friendship Hospital, Beijing, China; 2 Graduate School of Peking Union Medical College, Beijing, China

**Keywords:** IgA nephropathy, Immunosuppressive agents

## Abstract

**Background:**

Immunosuppressive agents have been widely used in the treatment of IgA nephropathy (IgAN), but the efficacy and safety remain controversial. The recent STOP-IgAN and TESTING studies have again focused attention on the application of immunosuppressive agents in IgAN. This study investigated the benefits and risks of immunosuppressive agents in IgAN.

**Methods:**

MEDLINE, EMBASE, the Cochrane Library, and article reference lists were searched for randomized controlled trials (RCTs) comparing immunosuppressive agents with any other non-immunosuppressive agents for treating IgAN. A meta-analysis was performed on the outcomes of proteinuria, creatinine (Cr), estimated glomerular filtration rate (eGFR), and adverse events in patients with IgAN, and trial sequential analyses were also performed for outcomes.

**Results:**

Twenty-nine RCTs (1957 patients) that met our inclusion criteria were identified. Steroids (weighted mean difference [WMD] –0.70, 95% confidence interval [CI] –1.2 to − 0.20), non-steroidal immunosuppressive agents (NSI) (WMD –0. 43, 95% CI − 0.55 to − 0.31), and combined steroidal and non-steroidal immunosuppressive agents (S&NSI) (WMD –1.46, 95% CI − 2.13 to − 0.79) therapy significantly reduced proteinuria levels compared with the the control group. Steroid treatment significantly reduced the risk of end-stage renal disease (ESRD) (relative risk [RR] 0.39, CI 0.19 to 0.79) compared with the control group. The immunosuppressive therapy group showed significant increases in gastrointestinal, hematological, dermatological, and genitourinary side effects, as well as impaired glucose tolerance or diabetes. Hyperkalemia was more common in the control group.

**Conclusion:**

Immunosuppressive therapy can significantly reduce proteinuria and ESRD risk in patients with IgAN, but with a concomitant increase in adverse reactions. Therefore, care is required in the application of immunosuppressive agents in IgAN.

## Background

IgA nephropathy (IgAN) is one of the most common primary glomerular diseases [1]. A systematic review demonstrated an overall population incidence of IgAN of 2.5/100000/year [2]. There is still no uniform standard of treatment for IgAN. The treatment of IgAN mainly includes reducing urinary protein and blood pressure (mainly with angiotensin-converting enzyme inhibitors (ACEIs) and angiotensin receptor blockers (ARBs)), glucocorticoid therapy, non-hormone immunosuppressive therapy, and other treatments. The 2012 Kidney Disease: Improving Global Outcomes (KDIGO) guidelines [3] for IgAN recommend treatment with a renin-angiotensin system (RAS) blocker, such as ACEIs and ARBs, in patients with proteinuria with protein excretion > 1 g/day. Corticosteroid therapy can be considered in patients with proteinuria > 1 g/day after 3–6 months of best supportive treatment and without renal failure. Intensive immunosuppression is reserved for patients with crescents in more than half the glomeruli and a rapid decline in renal function. In the past, we believed that the application of immunosuppressants could bring more benefits to IgAN patients with middle and high risk.

The publication of the Supportive versus Immunosuppressive Therapy of Progressive IgA Nephropathy (STOP-IgAN) trial in 2015 and Therapeutic Evaluation of Steroids in IgA Nephropathy Global (TESTING) trial in 2017 focused attention on the treatment of IgAN with immunosuppressive agents. According to the results of these two large randomized controlled trials (RCTs), there is still no clear evidence that immunosuppressive therapy can improve the prognosis of IgAN. Therefore, we retrieved RCTs on immunosuppressive therapy for IgAN, and performed a meta-analysis of the efficacy and safety of immunosuppressive therapy in this disease.

Immunosuppressive agents were divided into three subgroups for this meta-analysis: steroids, non-steroidal immunosuppressive (NSI) agents (NSI agents can be seen as steroid-sparing but not as steroid replacing agents), and steroids combined with non-steroidal immunosuppressive (S&NSI) agents. Their efficacy and safety were compared relative to controls for the treatment of IgAN.

This meta-analysis was performed in accordance with the recommendations of the Cochrane handbook for systematic reviews of interventions [4] and is reported in compliance with the Preferred Reporting Items for Systematic Reviews and Meta-Analyses (PRISMA) statement guidelines [5].

## Methods

### Inclusion and exclusion criteria

This investigation required studies to meet the following inclusion criteria: the study was an RCT; the study compared different immunosuppressive agents versus non-immunosuppressive agents/placebo/no treatment; and study subjects were adult or pediatric patients with biopsy-proven IgAN.

Studies were rejected according to the following exclusion criteria: immunosuppressant not given orally or intravenously (intestinal steroid budesonide was excluded though 20% bioavailable); study subjects with secondary IgAN; no data available for this study in the article, data included in other articles, or data repeated in other articles; and article not in English.

### Data sources and searches

The MEDLINE, EMBASE, and Cochrane Library medical databases were searched to retrieve relevant studies. Searches were performed in English, and each search retrieved studies that were published between establishment of the database and May 2018.

A comprehensive search strategy was established to ensure the comprehensive and accurate retrieval of studies. The MEDLINE and Cochrane Library databases were searched using the method described in the Cochrane Policy Manual [6], whereas EMBASE was searched using a sensitivity–specificity filter optimized [7]. The following search terms were used: IgAN, steroids, glucocorticoids, immunosuppressive agents, angiotensin-converting enzyme inhibitors, angiotensin receptor antagonists, and placebo. Furthermore, we also searched relevant professional journals manually.

### Data extraction and quality assessment

Two investigators (ZZ and YY) independently selected studies from the retrieved literature and extracted the data and analytical results. If the two investigators had different opinions about the quality of a study, a third investigator (SMJ) examined the controversial literature and discussed it with the two aforementioned investigators. Data were included only if the three authors achieve consensus regarding the data.

If necessary, daily proteinuria was recalculated as g/day. Values for eGFR were based on the data provided by the authors of the included studies.

We assessed treatment-related changes based on mean values and standard deviations (SDs) changes between the pre-treatment and post-treatment. As the standard error of the mean (SEM) was used in some studies, we calculated the SD using the formula: SEM × square root of sample size. In addition, 95% confidence intervals (CIs) were used in some studies; we calculated the SD using the formula: ((upper limit of 95% CI – lower limit of 95% CI)/(2 × 1.96)) × √(n). Assessment of the risks of publication bias followed the Cochrane handbook.

### Risk of bias assessment

Two authors (ZZ and YY) independently evaluated risk of bias using the Cochrane risk-of-bias tool [8]. We reviewed each trial and gave a score of bias according to the following criteria: random sequence generation, allocation concealment, blinding of participants and personnel, blinding of outcome assessment, incomplete outcome data, selective reporting, and other bias.

### Statistical analyses

To compare the effects of immunosuppressive agents and control treatment on proteinuria excretion and serum levels of creatinine, data on eGFR and end-stage renal disease (ESRD) were extracted for meta-analyses. Subgroup analyses were performed for each outcome based on the type of immunosuppressive agent.

For continuous outcomes, the differences in means and the 95% CI in mean change between baseline and end of treatment value were calculated for individual trials, and the weighted mean difference (WMD) was used as a summary estimator. Dichotomous outcome data from individual trials were analyzed using the relative risk (RR) measure and 95% CI. Heterogeneity of treatment effects between studies was investigated visually by examination of plots and statistically using the heterogeneity χ^2^ and I^2^ statistics. In all analyses, *P* < 0.05 was taken to indicate statistical significance. The fixed-effects and random-effects models were used for the meta-analysis of each indicator. Analyses were performed using Review Manager 5.2 (RevMan; Cochrane Collaboration, Oxford, UK).

### Trial sequential analyses

To assess whether the current meta-analysis had a adequate sample size to draw firm conclusions about the effects of interventions, we performed trial sequential analyses (TSAs) for outcomes. TSAs involves a cumulative meta-analysis to create a Z curve of the summarized observed effect and the monitoring boundaries for benefit and harm and estimate the optimal sample size [9]. A sufficient level of evidence for the anticipated intervention effect may have been reached when the cumulative z curve crosses the trial sequential monitoring boundary. And there is insufficient evidence to reach a conclusion, if the z curve crosses none of the boundaries and the required information size has not been reached. These analyses were performed using the software TSA version 0.9 Beta (Copenhagen Trial Unit, Copenhagen, Denmark).

## Results

### Basic information regarding the included studies

**Fig. 1:**
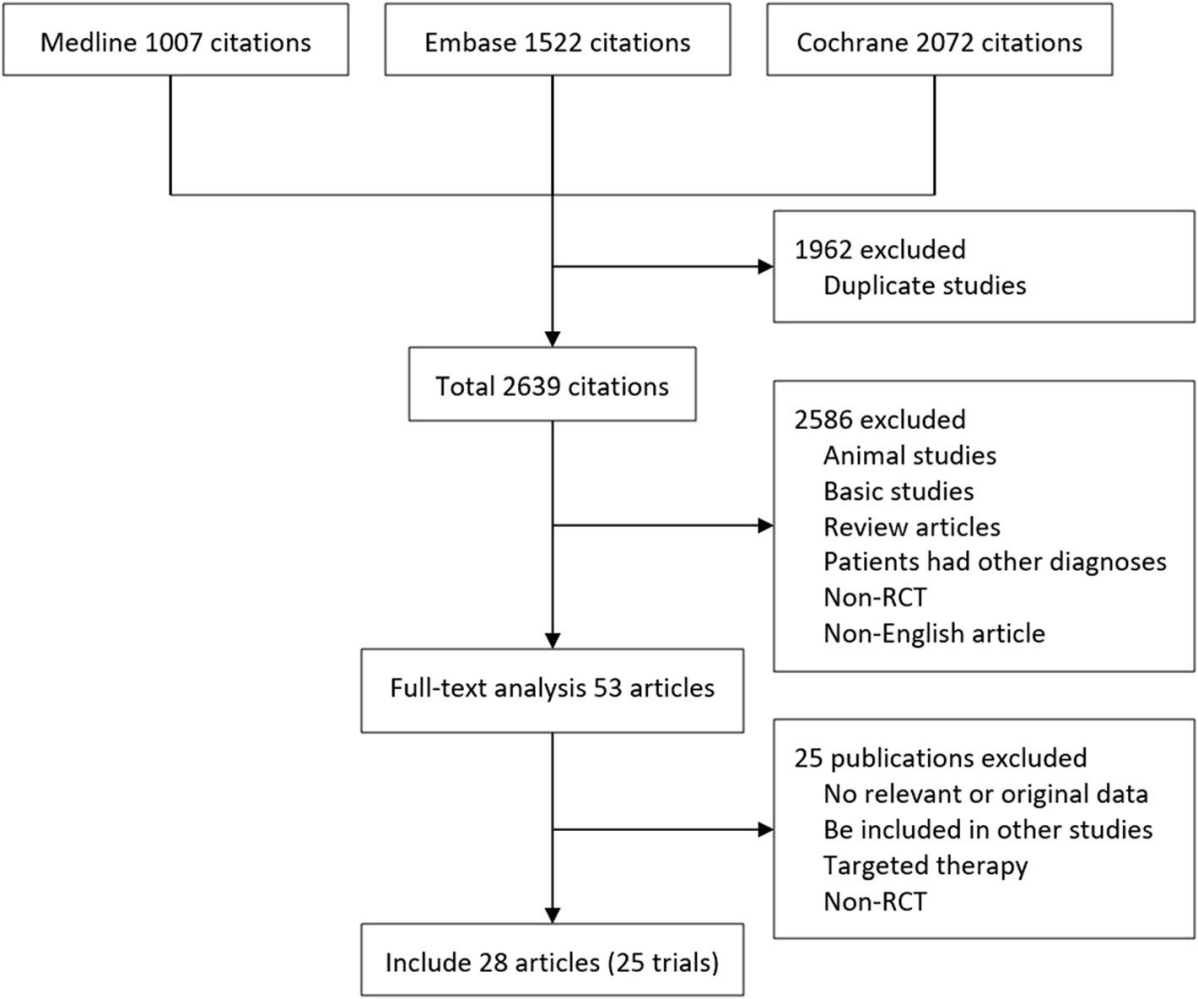
Results of systematic literature search on immunosuppressive treatment for IgAN

After performing electronic and manual searches, 4016 potentially relevant papers were obtained. After removing duplicated papers, 2639 papers remained. After browsing the titles and abstracts, 53 papers were selected. After reading the entire text of these 53 papers, 24 papers were excluded, and 28 papers describing 25 trials with a total of 1957 patients were ultimately included. The literature selection process is illustrated in Fig. 1, and detailed information regarding the examined studies is provided in Table 1 [10–38].

**Table 1:** Characteristics of RCTs included in the study

Study	Patient	Sample size	Intervention (treatment)	Intervention (control)	Follow-up
Ballardie 2002 [10]	18 to 54 years	38(19/19)	Prednisolone 40 mg/d (reduced to 10 mg/d by 2 year) + cyclophosphamide 1.5 mg/kg/day (adjusted down to the nearest 50 mg)	no immunosuppression	24 M
Cheng 2015 [11]	18–55 years old, hypertension under control, urinary proteins 0.5–3.5 g/24 h, Cr < 265.2 μmol/L	84(42/42)	leflunomide 20 mg/d + Valsartan	Valsartan	24 M
Cruzado 2011 [12]	18–70 years old, eGFR 30-60 ml/min/1.73m^2^, proteinuria > 1 g/d; BP > 140/90 mmHg with proteinuria 0.3-1 g/d	23(14/9)	SRL 1 mg/d (initial) + enalapril (or ACEI) + atorvastatin (or other statin)	Enalapril (or ACEI) + atorvastatin (or other statin)	12 M
Frisch 2005 [13]	18–75 years old, protein> 1 g/d	32(17/15)	MMF 1000 mg bid +ACEI/ARB	Placebo + ACEI/ARB	12 M
Harmankaya 2002 [14]	13–63 years, mean Ccr 89.2 ± 10.2 ml/min	43(21/22)	Prednisolone 40 mg/day + azathioprine 100 mg/day	no specific treatment	60 M
Hirai 2017 [15]	urinary protein excretion > 0.5 g/day, age > 16 years	42(21/21)	MZR 150 mg once daily orally in the morning for 12 months + Standard treatment	Standard treatment	36 M
Hogg 2015 [16]	7–70 years old; UPCR > 0.6 g/g (males) or > 0.8 g/g (females); eGFR> 50 mL/min/1.73 m2 (or > 40 mL/min/1.73 m2 in those already receiving ACE or ARB).	52(25/27)	MMF 25 to 36 mg/kg/d (Max dose of 1 g/d) + lisinopril	lisinopril or placebo 25 to 36 mg/kg/d (Max dose of 1 g/d)	12 M
Julian 1993 [17]	Ccr > 25 ml/min/1.73 m^2^	35(17/18)	prednisone	no placebo	12 M
Yoshikawa 1999^a^ [18]	< 15 Years old	78(40/38)	Prednisolone 2 mg/kg/d in three divided doses for a total dose of not more than 80 mg/d for 4w, followed by 2 mg/kg /2d, given as a single dose in the morning of every other day for 4w, 1.5 mg/kg/2d for 4w, and 1 mg/kg/2d for 21 m + azathioprine 2 mg/kg/d in a single morning dose for 24 m + heparin-warfarin + dipyridamole	heparin-warfarin + dipyridamole	24 M
Katafuchi 2003 [19]	≤60 years old, Cr < 1.5 mg/dl(132.6umol/L)	90(43/47)	prednisolone orally: 20 mg/d for 1 month, followed by 15 mg/d for 1 month, 10 mg/d for 1 month, 7.5 mg/d for 3 months, and 5 mg/d for 18 months + dipyridamole 150–300 mg/day	Dipyridamole 150–300 mg/day	60 M
Kim 2013 [20]	18–70 years old, serum creatinine ≤1.5 mg/dL or eGFR ≥45 ml/min/1.73 m^2^, UACR 0.3-3 g/g creatinine, BP < 130/80 mmHg	40(20/20)	Tacrolimus 0.1 mg/kg/day, 8 weeks (maintain trough levels at 5–10 ng/ml) → 0.05 mg/kg/day, 16 weeks (maintain the trough level in 5–10 ng/ml) + RASi(9/20)	RASi(11/20), placebo	16 W
Koike 2008 [21]	NA	48(24/24)	initially treated with 0.4 mg/kg/day of prednisolone (20–30 mg/day) for the first 4 weeks, and the dose was gradually reduced to 10–20 mg on alternate days for the next 12 months, and then 5–10 mg on alternate days for a subsequent year	Dipyridamole or dilazep hydrochloride	24 M
Pozzi 1999^b^ [22]	15–69 years old, urinary protein excretion of 1.0–3.5 g/d, Cr ≤ 133 umol/L (1.5 mg/dL)	86(43/43)	methylprednisolone intravenously for 3 consecutive days; this course was repeated 2 months and 4 months later. Oral prednisone was given at a dose of 0.5 mg/kg on alternate days for 6 months.	Supportive treatment	60 M
Lai 1986 [23]	14–42 years old, IgAN & NS	34(17/17)	prednisone/prednisolone 40-60 mg/d, reduce by half after 8 weeks	Supportive therapy	38 M
Lv 2009 [24]	18–65 years old, urinary proteins 1–5 g/d, eGFR> 30 ml/min	63(33/30)	prednisone: 0.8–1.0 mg/kg/day for 8 weeks, tapered by 5–10 mg every 2 weeks + cilazapril	cilazapril	48 M
Lv 2017 [25]	proteinuria> 1 g/d, eGFR: 20 -120 ml/min/1.73m^2^	262(136/126)	oral methylprednisolone (0.6–0.8 mg/kg/d; maximum, 48 mg/d)	placebo	60 M
Maes 2004 [26]	> 18 years old, inulin clearance 20–70 mL/min/1.73m^2^, proteinuria > 1 g/day, BP > 140/90 mmHg,	34(21/13)	MMF: 2 g/d + ACEI	Placebo (identical lactose-containing capsules)	36 M
Manno 2009 [27]	16–70 years old, proteinuria> 1 g/d, eGFR≥50 ml/min/1.73m^2^	97(48/49)	prednisone: 1.0 mg/kg/day (Max: 75 mg/day) for 2 months, tapered by 0.2 mg/kg/day every month ramipril	ramipril	5Y
Rauen 2015 [28]	proteinuria> 0.75 g/d after 6 months support treatment	162(82/80)	Supportive Care (100%) + Immunosuppression	RASi (77/80)	36 M
Shoji 2000 [29]	15–55 years old, proteinuria less than 1.5 g/d, serum creatinine level less than 1.5 mg/dL	19(11/8)	prednisolone 0.8 mg/kg of body weight; this was gradually reduced to a daily dose of 0.4 mg/kg of body weight during the first month of therapy, and then tapered to 10 mg very other day for the remainder of the 1 year of therapy	Dipyridamole 300 mg/day	12 M
Tang 2005^c^[30]	urinary proteins> 1 g/d, BP < 125/85 mmHg, Cr < 300umol/L(3.4 mg/dl)	40(20/20)	MMF 2 g/day (weight ≥ 60 kg), 1.5 g/day (weight < 60 kg) + ACEI/ARB(16:4)	ACEI/ARB (14:6)	72 W
Walker 1990 [31]	24 h pro> 1.0 g/d, 120umol/L < Cr < 200umol/L one or more	52(25/27)	Cyclophosphamide (1–2 mg/kg/24 h - maximum of 100 mg/24 h and ajusted according to peripheral white cell counts) + dipyridamole +warfarin	no treatment	2Y
Wu 2016 [32]	18–55 years, proteinuria of 0.5–3.5 g/d, serum creatinine < 265 μmol/L, blood pressure between 90/60 and 130/80 mmHg	399(100/299)	Leflunomide 20 mg/d + telmisartan + clopidogrel placebo	Telmisartan + Leflunomide placebo + clopidogrel placebo & Telmisartan + clopidogrel + Leflunomide placebo & Telmisartan + clopidogrel	24w
Xie 2011 [33]	14–70 years old, urinary protein excretion: 0.5 to 3.5 g/24 h, Cr < 353.6 umol/L	64(34/30)	MZR 200 mg/d (weight < 50 kg), 250 mg/d (weight > 50 kg), 150 mg/d (Cr > 176.8 umol/L) + losartan	Losartan	12 M
Woo 1987 [34]		48(27/21)	cyclophosphamide 1.5 mg/kg per day+ dipyridamole + warfarin	No treatment	36 M

*Abbreviations*: *NA* not applicable, *MMF* mycophenolate mofetil, *SRL* sirolimus, *MZR* mizoribine

^a^Kamei 2011 and Yoshikawa 1999 describe the same trial, but the available data provided by the articles are different. Here, only the data of Yoshikawa 1999 are listed

^b^Locatelli 2001 and Pozzi 2004 were follow-up studies of Pozzi 1999, and only the data of Pozzi 1999 are listed here

^c^Tang 2010 was a follow-up study of Tang 2005, and only the data of Tang 2005 are listed here

### Quality of trials

**Fig. 2:**
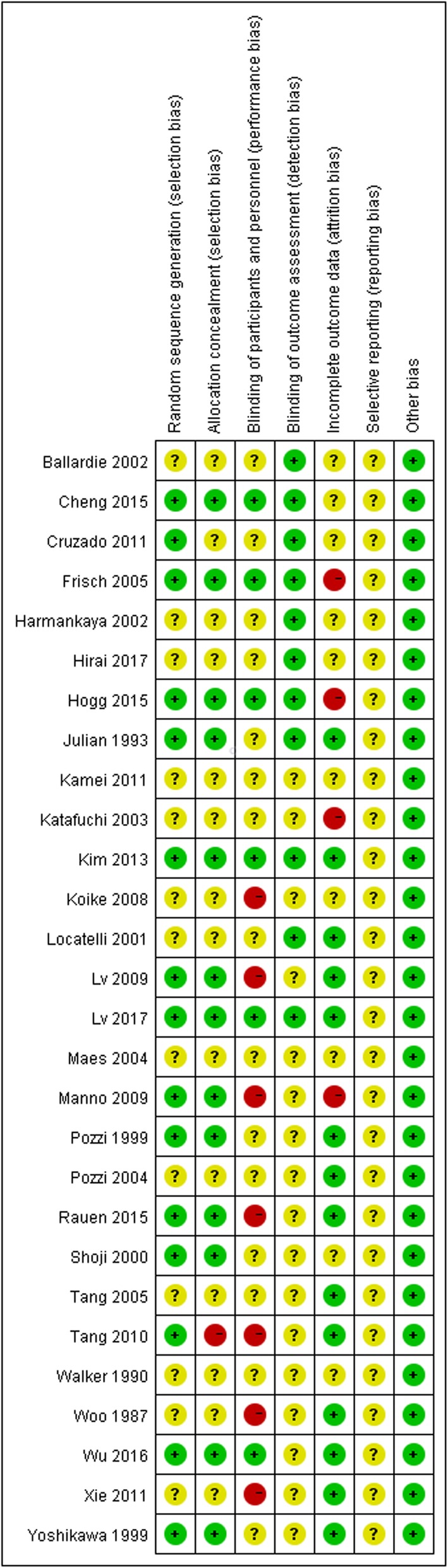
Risk of bias graph

By current standards, reporting of key indicators of trial quality was suboptimal. Some studies in particular provided few details on the process of randomization and concealment of allocation. Only six studies were double-blinded trials. Seven studies used an open-label design. The bias and overall risk diagrams of the included studies are presented in Fig. 2.

### Effects on proteinuria

**Fig. 3:**
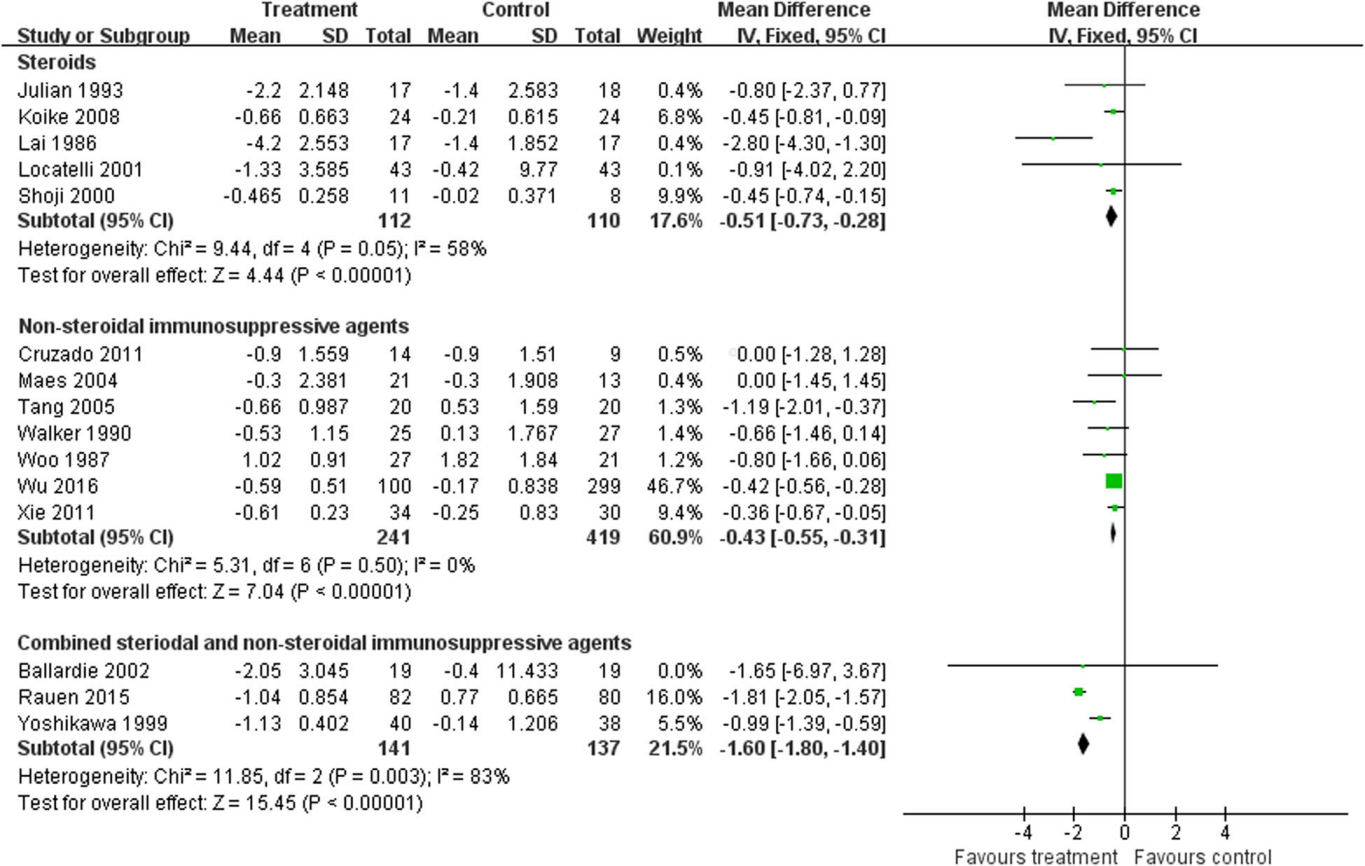
Proteinuria: Effects of immunosuppressive agents on proteinuria in patients with IgAN. CI, confidence interval

The difference in the means of urinary protein excretion between end of treatment and baseline was significantly lower in the steroid group than in controls (five trials [17, 21, 23, 29, 36], 222 patients; WMD –0.51, 95% CI − 0.73 to − 0.28, with a fixed-effects model; WMD –0.70, 95% CI − 1.2 to − 0.20, with a random-effects model; I^2^ = 58%; Fig. 3). After removing Lai [23], heterogeneity I^2^ changed to 0.

Patients receiving NSI alone showed a more significant reduction of urinary protein excretion after treatment compared to controls (seven trials [12, 26, 30–34], 660 patients, WMD –0.43, 95% CI − 0.55 to 0.31, with a fixed-effects model; WMD –0. 43, 95% CI − 0.55 to − 0.31, with a random-effects model; I^2^ = 0; Fig. 3).

With the S&NSI treatment approach, patients had a more significant reduction of urinary protein excretion after treatment compared to controls (three trials [10, 18, 28], 278 patients, WMD –0.16, 95% CI − 1.8 to − 1.4, I^2^ = 83%, with a fixed-effects model; WMD –1.42, 95% CI − 2.18 to − 0.66, I^2^ = 89%, with a random-effects model; Fig. 3). After removing Yoshikawa [18], heterogeneity I^2^ changed to 0.

**Fig. 4:**
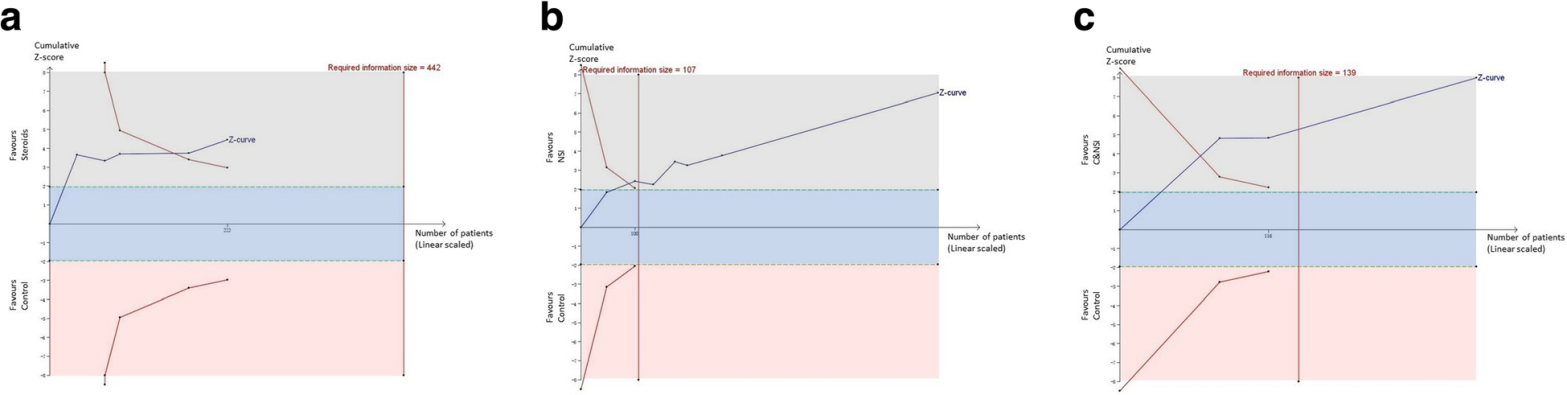
Trial sequential analyses of proteinuria. **a** Five comparisons between steroids and controls. **b** Seven comparisons between NSI and controls. **c** Three comparisons between S&NSI and controls. Effects on renal function and renal survival

TSAs of steroids, NSI, and S&NSI all indicated that the cumulative z curve crossed both the conventional boundary and the trial sequential monitoring boundary (Fig. 4).

### Creatinine

**Fig. 5:**
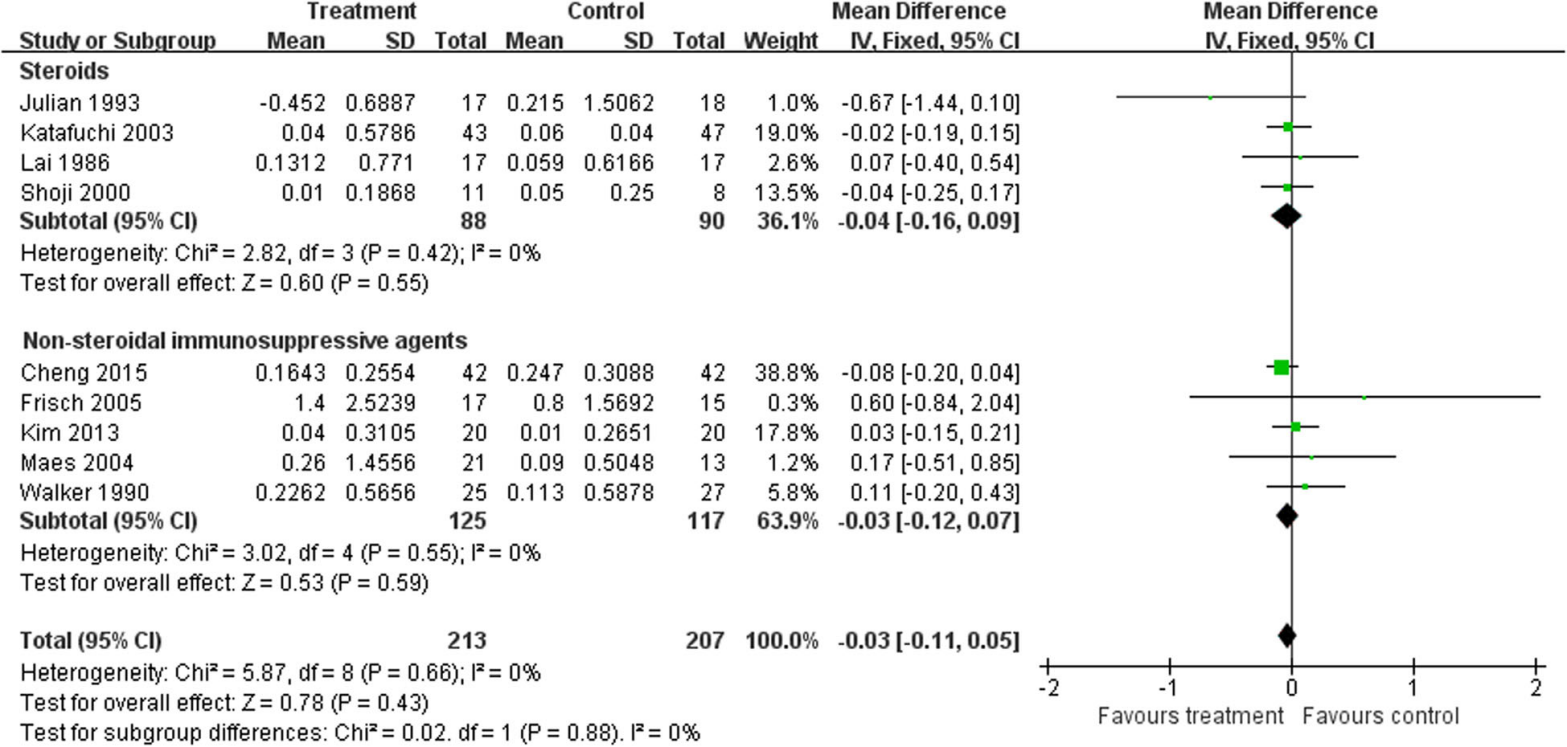
Creatinine: Effects of immunosuppressive agents on creatinine levels in patients with IgAN. CI, confidence interval

There were no statistically significant differences in creatinine changes between baseline and end of treatment between immunosuppressive treatment and control groups (nine trials [11, 13, 17, 19, 20, 23, 26, 29, 31], 420 patients, WMD –0.03, 95% CI − 0.11 to 0.15, with a fixed-effects model; WMD –0.03, 95% CI − 0.11 to 0.05, with a random-effects model; I^2^ = 0%; Fig. 5).

TSAs of nine comparisons illustrated that the cumulative z curve did not cross the conventional boundary or the line of required information size, indicating that the evidence was insufficient. Therefore, further trials are required.

### eGFR

**Fig. 6:**
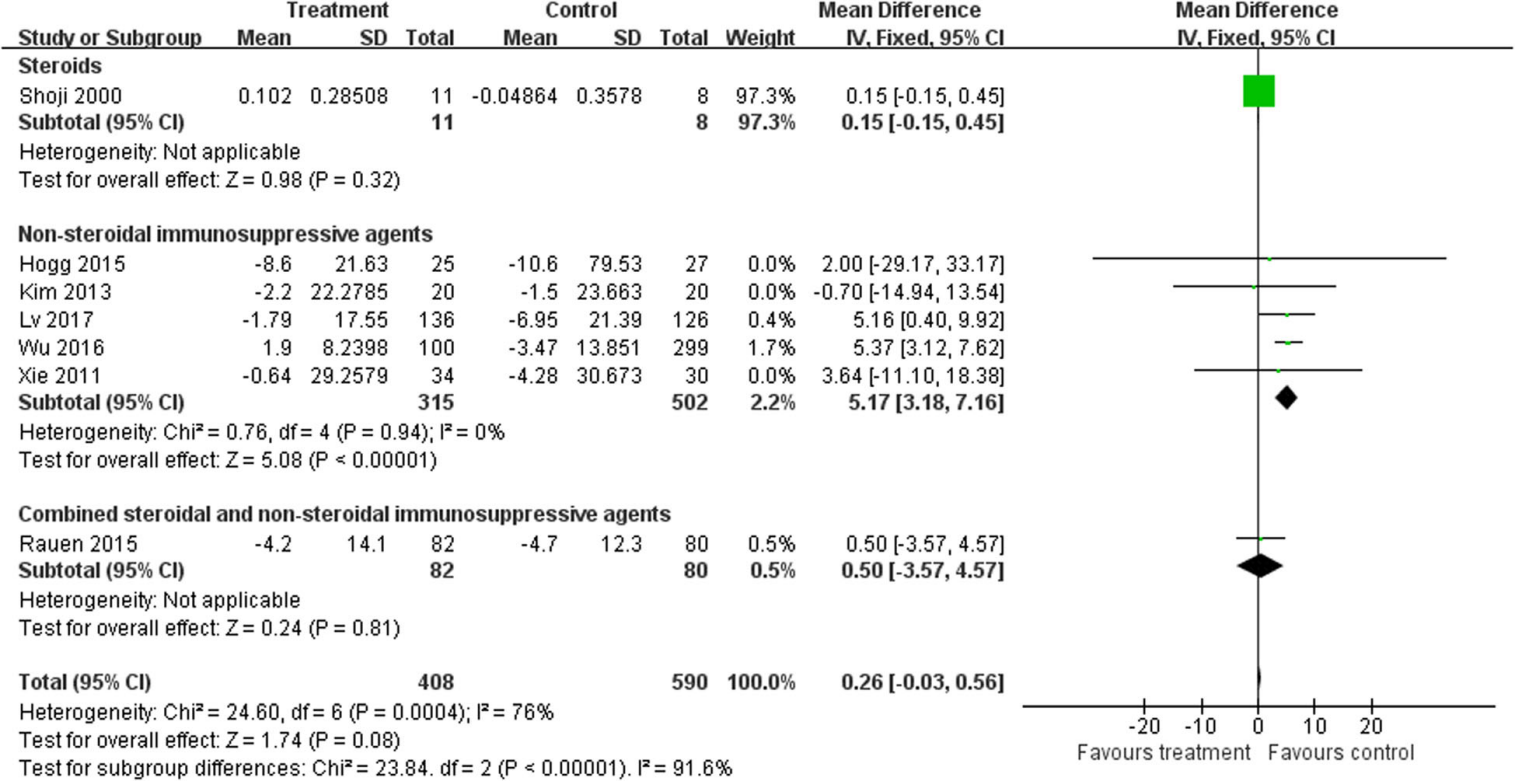
eGFR: Effects of immunosuppressive agents on estimated glomerular filtration rate in patients with IgAN. CI, confidence interval

The differences in the means of eGFR between end of treatment and baseline were significantly higher in the NSI group than in controls (five trials [16, 20, 25, 32, 33], 817 patients; WMD 5.17, 95% CI 3.18 to 7.16, with a fixed-effects model; WMD 5.17, 95% CI 3.18 to 7.16, with a random-effects model; I^2^ = 0%; Fig. 6). TSAs of five comparisons indicated that the cumulative z curve crossed the conventional boundary, but did not cross the trial sequential monitoring boundary.

However, when the steroid and S&NSI groups were added, there were no significant differences in eGFR changes in immunosuppressive treatment compared to controls (seven trials [16, 20, 25, 28, 29, 32, 33], 998 patients, WMD 0.26, 95% CI − 0.03 to 0.56, with a fixed-effects model; WMD 2.52, 95% CI − 0.49 to 0.53, with a random-effects model; I^2^ = 76%; Fig. 6). TSAs of seven comparisons indicated that the cumulative z curve did not cross the conventional boundary or the line of required information size.

### ESRD

**Fig. 7:**
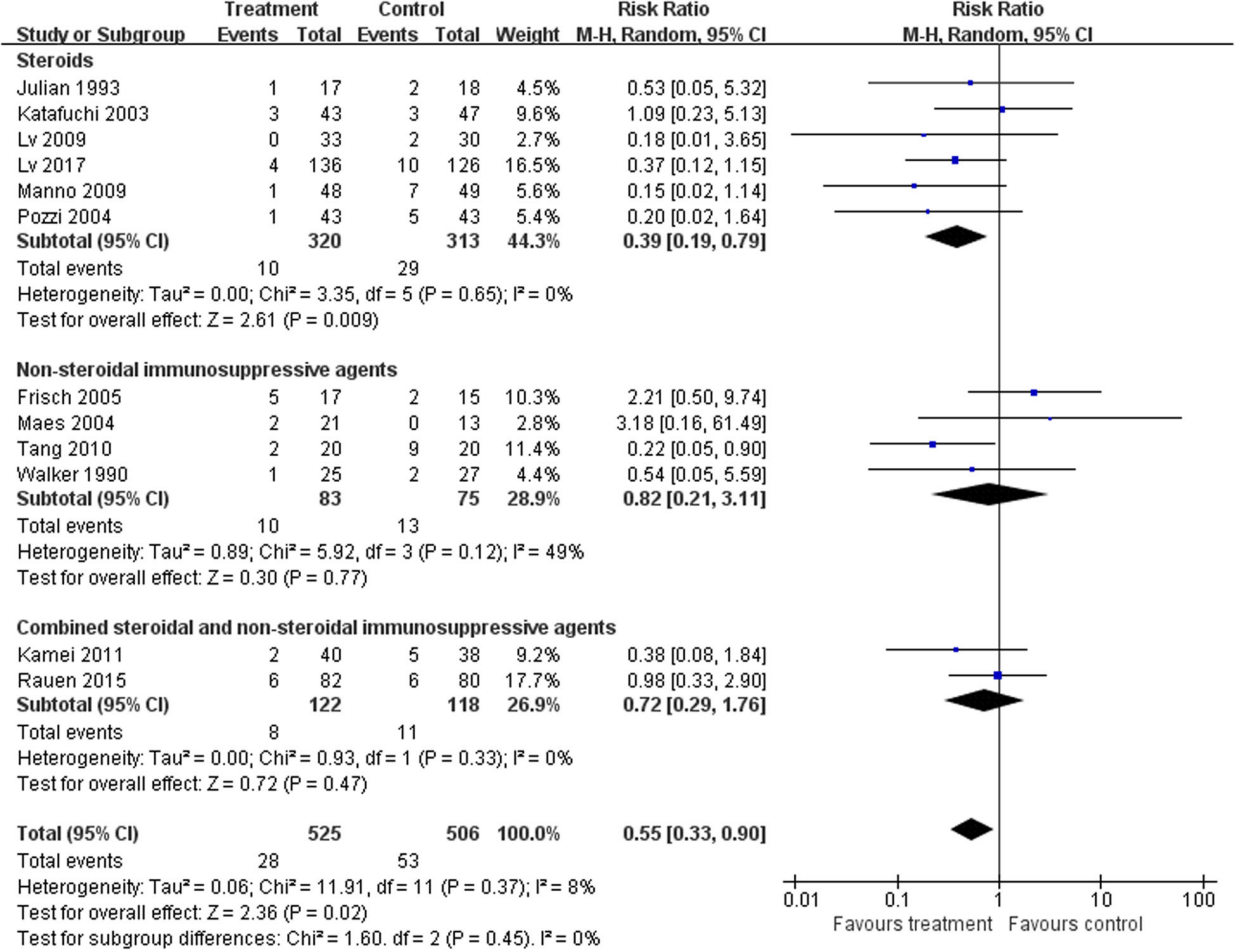
ESRD: Effects of immunosuppressive agents on end-stage renal disease in patients with IgAN. CI, confidence interval; RR, relative risk

There was a lower risk of reaching ESRD in the immunosuppressive treatment group than in controls (12 trials [13, 17, 19, 24–28, 31, 35, 37, 38], 1031 patients; RR 0.51, 95% CI 0.33 to 0.08, with a fixed-effects model; RR 0.55, 95% CI 0.33–0.90, with a random-effects model; I^2^ = 8; Fig. 6). These analyses were dominated by the steroid treatment group (Fig. 7).

TSAs of steroids indicated that the cumulative z curve crossed both the conventional boundary and the trial sequential monitoring boundary.

### Adverse events of treatment

A total of 20 articles reported adverse events during the observation period. The types of adverse events varied widely, and included infection, cardiovascular disease, respiratory disease, hepatotoxicity, and many others; the 13 most commonly reported are listed in Table 2. As the number of infections reported in Rauen [28] was greater than the total number, RR could not be calculated for infections. TSAs of infection, gastrointestinal disease, hematological disease, dermatological disease, impaired glucose tolerance or diabetes mellitus, and hyperkalemia indicated that the cumulative z curve crossed the conventional boundary but did not cross the trial sequential monitoring boundary. In addition, TSAs of the other seven diseases indicated that the cumulative z curve did not cross the conventional boundary or the line of required information size.

**Table 2:** Main adverse events reported in the included RCTs

Main adverse events	No. of studies	Immunosuppressive agent group	Control group	RR (95% CI)		*P* value	
				FE	RE	FE	RE
Gastrointestinal	11	38/431	8/606	2.53 [1.15, 5.55]	2.42[1.07, 5.45]	0.02	0.03
Hematologic	9	16/373	6/551	2.17 [1.00, 4.68]	2.0[0.84, 4.77]	0.05	0.12
Dermatologic	7	16/273	3/463	4.09 [1.57, 10.66]	3.88[1.41, 10.64]	0.004	0.009
Hepatotoxicity	7	21/455	19/636	1.26 [0.72, 2.22]	1.26[0.70, 2.24]	0.42	0.44
Respiratory	6	9/371	12/544	0.81 [0.37, 1.74]	0.82[0.37, 1.82]	0.58	0.62
Infection	6	189/373	114/547	Not estimable	Not estimable	Not estimable	Not estimable
Impaired glucose tolerance or diabetes mellitus	5	15/326	5/316	2.61 [1.04, 6.55]	2.16[0.77, 6.05]	0.04	0.14
Elevation of blood pressure	4	14/193	16/389	0.96 [0.52, 1.79]	0.97[0.43, 2.22]	0.9	0.95
Malignant	4	4/167	2/157	1.40 [0.39, 4.98]	1.33[0.30, 5.93]	0.61	0.71
Musculoskeletal	3	5/238	3/226	1.47 [0.44, 4.93]	1.37[0.40, 4.71]	0.53	0.62
Hyperkalemia	3	2/156	11/350	0.23 [0.07, 0.71]	0.3[0.05, 1.98]	0.01	0.21
Genitourinary	3	6/59	0/56	4.59 [0.85, 24.85]	4.07[0.71, 23.39]	0.08	0.12
Death	2	3/218	2/206	1.42 [0.24, 8.44]	1.41 [0.23, 8.55]	0.70	0.71

*RR* relative risk, *CI* confidence intervals, *FE* fixed effect model, *RE* random effect model

## Discussion

Farnsworth [39] and Barnett [40] first used corticotropin between 1949 and 1950 for the treatment of lipoid nephrosis, which is now known as minimal change disease or childhood nephrotic syndrome. Chasis et al. [41] used nitrogen mustard to treat chronic glomerulonephritis and achieved good initial results, thus pioneering the use of immunosuppressive agents for the treatment of nephropathy. Immunosuppressive agents have been used for the treatment of kidney diseases for about 70 years. However, the outcomes immunosuppressive therapy for IgAN are controversial. Therefore, we included 28 reports published between 1986 and 2017 in a meta-analysis of the efficacy and safety of immunosuppressive treatment and control treatment in IgAN.

### Alleviation of proteinuria

Previous studies have suggested that treatment with steroids or alkylating agents can significantly reduce proteinuria levels in patients with IgAN [42–44]. Our meta-analysis also showed that immunosuppressive agents can significantly reduce the level of proteinuria. The levels of proteinuria in groups treated with steroids, NSI, or S&NSI were significantly reduced compared to controls. The heterogeneity of the steroid group was mainly derived from Lai [23], in which the inclusion criterion included nephrotic syndrome. In addition, the heterogeneity of the S&NSI group was mainly derived from Yoshikawa [18], in which the inclusion criterion included age < 15 years. Sequential analyses showed that immunosuppressive agents were effective for relieving proteinuria, and no additional sample size was required.

### Reducing the risk for ESRD

Our results suggest that non-steroidal immunosuppressive therapy may have a positive effect on eGFR. However, sequential analyses suggested that this is still inconclusive and further studies are required for confirmation. In addition, the treatment group showed a greater reduction in the risk for ESRD than the control group, and this effect was mainly due to the steroid treatment group. Sequential analyses showed that steroids could reduce the risk for ESRD without the need for a larger sample size. A relevant study [43] also suggested that high-dose short-course steroid therapy has a significant protective effect on renal function, while a low-dose long-course of steroids does not. Further studies are required to determine whether NSI or S&NSI can reduce the risk for ESRD.

### More adverse events

The use of immunosuppressive agents is often accompanied by side effects. The immunosuppressive therapy group showed significant increases in gastrointestinal, hematological, dermatological, and genitourinary side effects, as well as impaired glucose tolerance or diabetes in this meta-analysis. As the number of infection events reported in the STOP study was too high, even exceeding the total number of patients, it was not possible to calculate the RR value. However, across all studies, the proportion of infections reported was still higher in the immunosuppressive therapy group than in controls. In addition, the TESTING study had to be discontinued because of the excessive number of serious adverse events, mostly infections. By contrast, hyperkalemia was more common in the control group, which may have been related to the application of ACEI and ARB. However, it should be noted that sequential analyses indicated that the statistical results of the above adverse events should be verified by further experiments. Although there were more adverse events in the immunosuppressant group, immunosuppressive agents should be used when necessary because they significantly reduce the risk of ESRD (which means fewer dialysis and transplantation).

### Strengths and limitations

Our study had several limitations that should be taken into consideration. The results of bias analyses indicated that nearly half of the studies did not explicitly report the methods used for randomization. In addition, few studies used blinded methodologies. The quality of the reports in the literature is unsatisfactory. In addition, there were some differences in the inclusion criteria between each study, such as age, proteinuria level, and renal function, and these confounding factors led to a high degree of data heterogeneity. Our results show that glucocorticoids therapy has no significant effect on serum creatinine or eGFR in patients with IgA nephropathy. However, because chronic administration of glucocorticoids significant muscle loss and endogenous creatinine production can ocurr, possible errors in estimation of GFR using serum creatinine based formulas [45] may have led to an over optimistic conclusion.

## Conclusions

In conclusion, the importance of a meta-analysis of the use of immunosuppressant in the treatment of IgA nephropathy was noted. The use of immunosuppressants in the treatment of IgA nephropathy has been shown to significantly reduce proteinuria. Sequential analyzes showed that immunosuppressive agents were effective for relieving proteinuria, and no additional sample size was required. In addition, immunosuppressants significantly decrease the risk for ESRD. Immunosuppressant therapy of IgAN has significant benefits. But it also increases the risk for serious adverse reactions. Therefore, in the course of using immunosuppressive agents, close observation should be carried out to prevent and control complications. In addition, further well-designed and high-quality RCTs are needed to explore the applicability and optimal methods of immunosuppressant treatment.

## Data Availability

All data generated or analysed during this study are included in this published article.

## Abbreviations

ACEIs: Angiotensin-converting enzyme inhibitors
ARBs: Angiotensin II receptor blockers
CIs: Confidence intervals
ESRD: End-stage renal disease
IgAN: IgA nephropathy
KDIGO: Improving Global Outcomes
NSI: Non-steroidal immunosuppressive
RAS: Renin-angiotensin system
RCTs: Randomized controlled trials
RR: Relative risk
S&NSI: Non-steroidal immunosuppressive
SDs: Standard deviations
SEM: Standard error of the mean
TSAs: Trial sequential analyses
WMD: Weighted mean difference
